# Supplementary material for: Designable Poly(methacrylic Acid)/Silver Cluster Ring Arrays as Reflectance Spectroscopy-Based Biosensors for Label-Free Plague Diagnosis
Source: Polymers (Basel). 2023 Apr 17;15(8):1919. doi: 10.3390/polym15081919 (PMC10143817; doi:10.3390/polym15081919)
Supplement: Supplementary file 1 [file polymers-15-01919-s001.zip › polymers-2305845-supplementary.docx]

Supplementary Materials

Designable Poly(methacrylic Acid)/Silver Cluster Ring Arrays as Reflectance Spectroscopy-Based Biosensors for Label-Free Plague Diagnosis

Chih-Wei Chen ^1,2,3^, Shih-Hsun Chen ^4^, Chih-Feng Huang ^5,^* and Jem-Kun Chen ^3,^*

| **Citation:** Chen, C.-W.; Chen, S.-H.; Huang, C.-F.; Chen, J.-K. Designable Poly(methacrylic Acid)/Silver Cluster Ring Arrays as Reflectance Spectroscopy-Based Biosensors for Label-Free Plague Diagnosis. *Polymers* **2023**, *14*, x. https://doi.org/10.3390/xxxxx  Academic Editor(s): Dimitrios Bikiaris  Received: 10 March 2023  Revised: : 12 April 2023  Accepted: 13 April 2023  Published: date  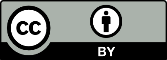  **Copyright:** © 2023 by the authors. Licensee MDPI, Basel, Switzerland. This article is an open access article distributed under the terms and conditions of the Creative Commons Attribution (CC BY) license (https://creativecommons.org/licenses/by/4.0/). |
| --- |

^1^ Division of Neurosurgery, Department of Surgery, Chi Mei Medical Center, Tainan 710, Taiwan

^2^ Department of Occupational Safety and Health/Institute of Industrial Safety and Disaster Prevention, College of Sustainable Environment, Chia Nan University of Pharmacy and Science, Tainan 717, Taiwan

^3^ Department of Materials and Science Engineering, National Taiwan University of Science and Technology, 43, Sec. 4, Keelung Road, Taipei, 106, Taiwan, ROC

^4^ Department of Mechanical Engineering, National Yang Ming Chiao Tung University, No. 1001, Daxue Rd. East Dist., Hsinchu City 300093, Taiwan

^5^ Department of Chemical Engineering, i-Center for Advanced Science and Technology (iCAST), National Chung Hsing University, Taichung 40227, Taiwan;

* Correspondence: huangcf@dragon.nchu.edu.tw (C.-F. H.); jkchen@mail.ntust.edu.tw (J.-K. C.); Tel.: +886-2-27376523. (J.-K. C.) Fax: +886-2-27376544 (J.-K. C.)

(a)

(b)

Figure S1: (**a**)TEM image and (**b**) DLS data of AgCs.
